# Supplementary figures and images for: Epidemiology of giardiasis and assemblages A and B and effects on diarrhea and growth trajectories during the first 8 years of life: Analysis of a birth cohort in a rural district in tropical Ecuador
Source: PLoS Negl Trop Dis. 2023 Nov 20;17(11):e0011777. doi: 10.1371/journal.pntd.0011777 (PMC10695370; doi:10.1371/journal.pntd.0011777)

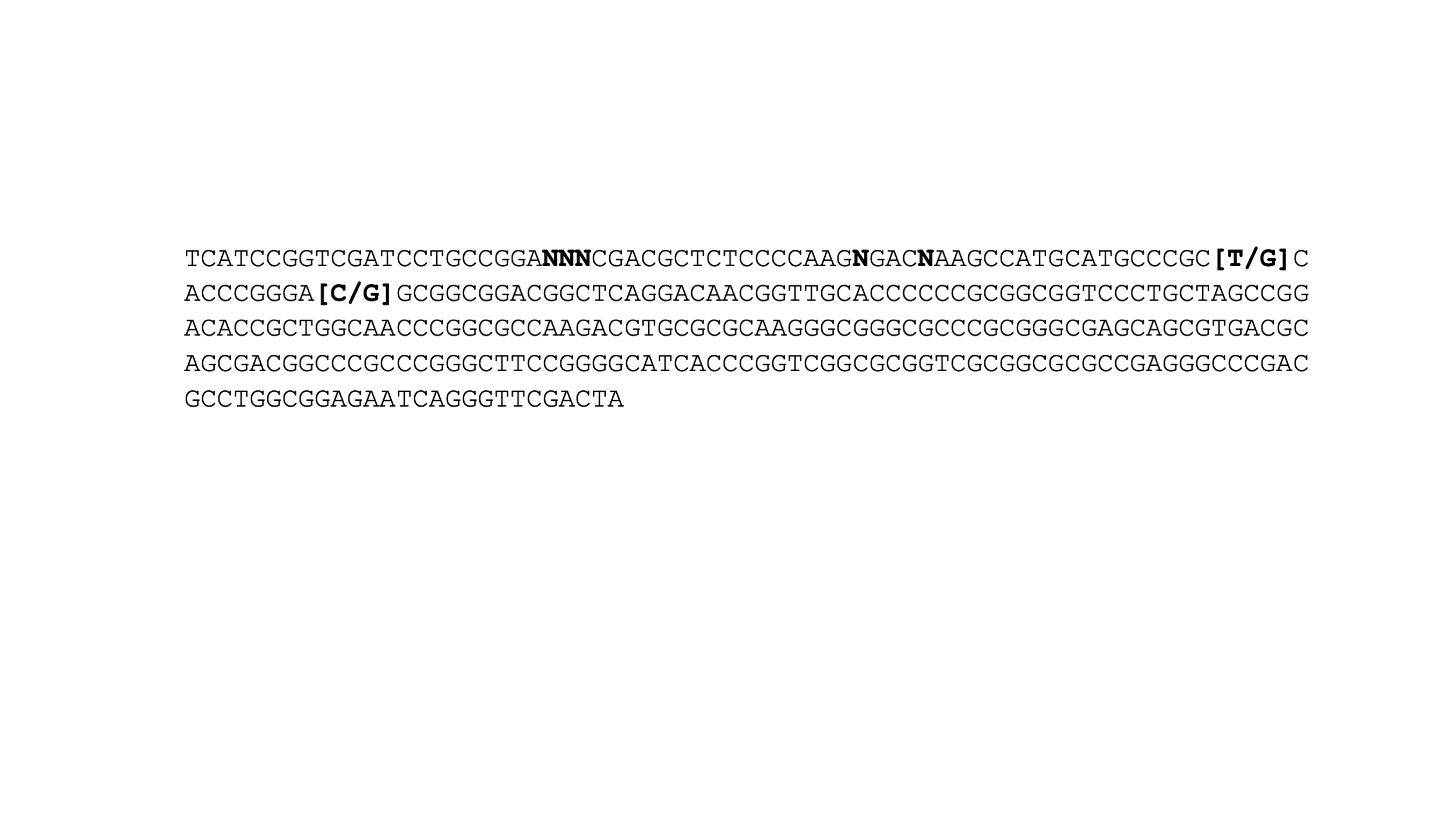

Supplement: S1 Fig — N’s correspond to SNPs and MNP’s masked and not targeted. (TIFF) [file pntd.0011777.s001.tiff]

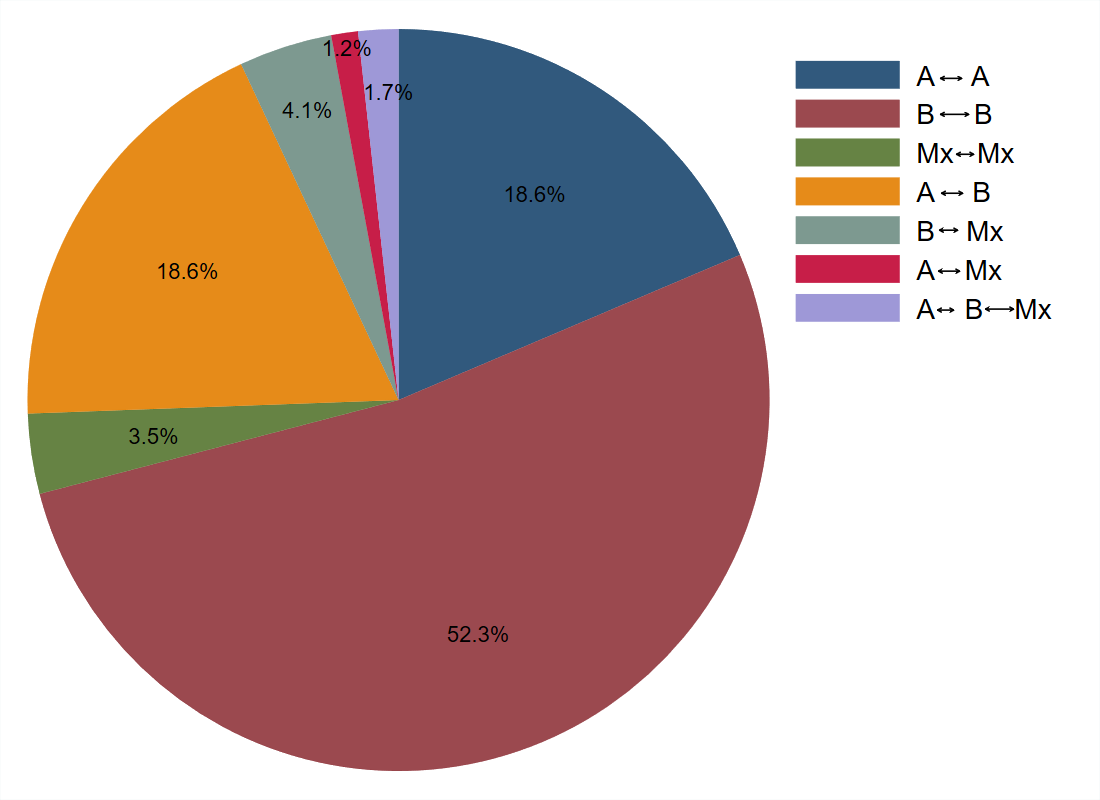

Supplement: S2 Fig — Shown are proportions that during follow-up have assemblages A (A) or (B) alone detected, assemblages A and B alone detected at different times during follow-up (A-B), and those with mixed and non-mixed infections (Mixed). (TIF) [file pntd.0011777.s002.tif]

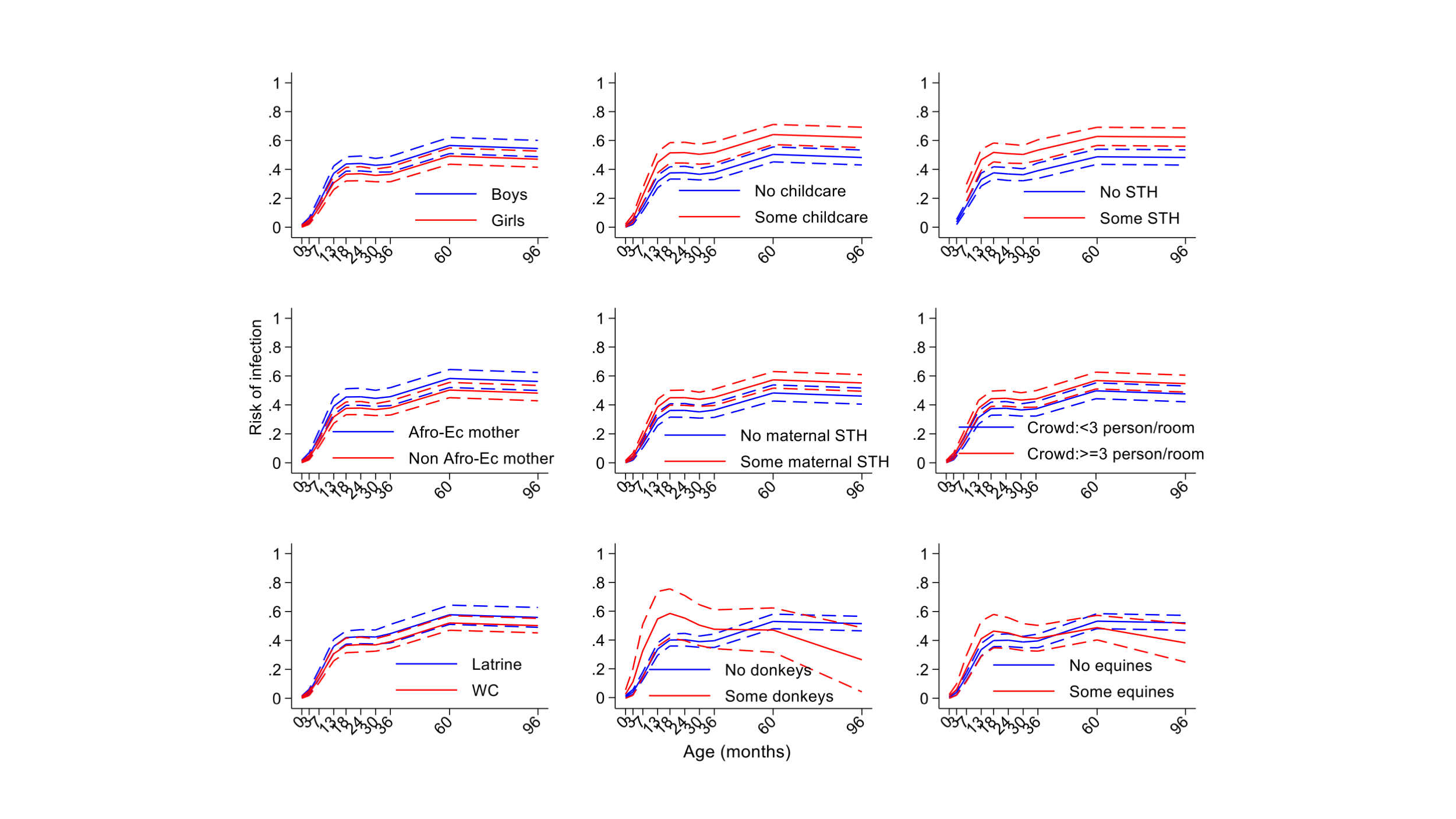

Supplement: S3 Fig — (TIFF) [file pntd.0011777.s003.tiff]

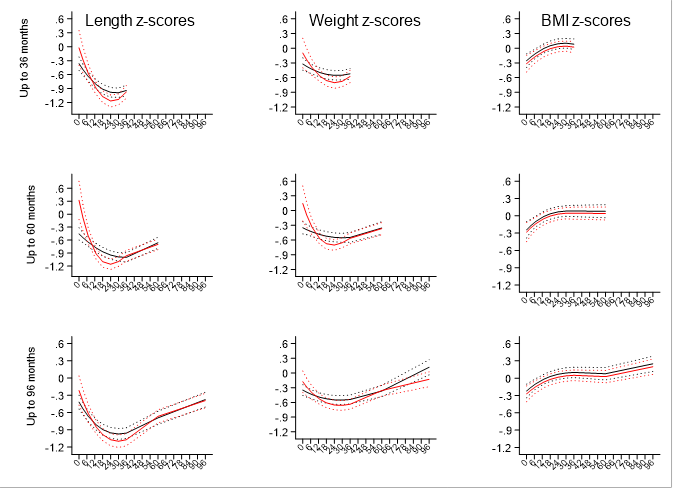

Supplement: S4 Fig — Y axes show z scores for each of the growth parameters. BMI–body mass index. Interrupted curves represent 95% confidence intervals. (TIFF) [file pntd.0011777.s004.tiff]
