# Supplementary material for: Epidemiology of giardiasis and assemblages A and B and effects on diarrhea and growth trajectories during the first 8 years of life: Analysis of a birth cohort in a rural district in tropical Ecuador
Source: PLoS Negl Trop Dis. 2023 Nov 20;17(11):e0011777. doi: 10.1371/journal.pntd.0011777 (PMC10695370; doi:10.1371/journal.pntd.0011777)
Supplement: S1 Table — (DOCX) [file pntd.0011777.s005.docx]

| Loci | Molecular analyses | Primer/ probe (reporter dye) | Primer or probe sequence (5′-3′) | Reference |
| --- | --- | --- | --- | --- |
| 16S rRNA | qPCR | Forward | CATGCATGCCCGCTCA | (Mejia et al., 2013)  [29] |
|  |  | Reverse | AGCGGTGTCCGGCTAGC |  |
|  |  | Probe (FAM) | AGGACAACGGTTGCAC |  |
| SSU rRNA | PCR for sequencing | Forward RH11 | CATCCGGTCGATCCTGCC | (Hopkins et al., 1997)  [33] |
|  |  | Reverse RH4 | AGTCGAACCCTGATTCTCCGCCAGG |  |
| SSU rRNA | 18S rRNA SNP Genotyping Assay | Forward | CCGGTCGATCCTGCCG | Present study |
|  |  | Reverse | GCAACCGTTGTCCTGAGC |  |
|  |  | Probe (VIC) | ATGCCCGCTCACCCG |  |
|  |  | Probe (FAM) | CCCGCGCACCCG |  |

**S1 Table. Sequences of primers and probes used in molecular analyses.**
