# Supplementary material for: Epidemiology of giardiasis and assemblages A and B and effects on diarrhea and growth trajectories during the first 8 years of life: Analysis of a birth cohort in a rural district in tropical Ecuador
Source: PLoS Negl Trop Dis. 2023 Nov 20;17(11):e0011777. doi: 10.1371/journal.pntd.0011777 (PMC10695370; doi:10.1371/journal.pntd.0011777)
Supplement: S2 Table — (DOCX) [file pntd.0011777.s006.docx]

| Age(months) | Raw data | Estimated prop | 95%CI -low | 95%CI - high | Denominator | Number of infected |
| --- | --- | --- | --- | --- | --- | --- |
| 1 | .0098 | .007711 | .0008704 | .0145516 | 102 | 1 |
| 3 | .0273 | .0400015 | .0232255 | .0567775 | 256 | 7 |
| 7 | .1481 | .1519878 | .1238468 | .1801287 | 351 | 52 |
| 13 | .3487 | .338309 | .2952289 | .3813891 | 347 | 121 |
| 18 | .292 | .4000955 | .3588119 | .4413791 | 137 | 40 |
| 24 | .4101 | .4023471 | .3589925 | .4457016 | 317 | 130 |
| 30 | .3692 | .3908884 | .3524904 | .4292864 | 195 | 72 |
| 36 | .3989 | .4004022 | .353666 | .4471384 | 376 | 150 |
| 60 | .5393 | .5265293 | .4765759 | .5764827 | 356 | 192 |
| 96 | .504 | .5062107 | .4561932 | .5562281 | 375 | 189 |

**S2 Table. Observed and predicted risk of G. lamblia infection by age.**
