# Supplementary material for: Epidemiology of giardiasis and assemblages A and B and effects on diarrhea and growth trajectories during the first 8 years of life: Analysis of a birth cohort in a rural district in tropical Ecuador
Source: PLoS Negl Trop Dis. 2023 Nov 20;17(11):e0011777. doi: 10.1371/journal.pntd.0011777 (PMC10695370; doi:10.1371/journal.pntd.0011777)
Supplement: S3 Table — Age-adjusted analyses include polynomial terms for age up to power of 5 (all P<0.001). RR–relative risk. CI–confidence interval. Time varying (tv) variables. Data on household factors were collected around the time of birth of the child unless specified as tv. Material goods–number of household electrical goods. Pigs, chickens cows, and equines–keeping these animals around house. Agriculture–child lives on a farm or visits a farm at least once a week. STH–soil-transmitted helminth infection. Non-Afro–non-Afro-Ecuadorian. yrs–years. m–months. SES -socioeconomic status. Overcrowding–persons/sleeping room. Statistically significant findings (P<0.05) are shown in bold. (DOCX) [file pntd.0011777.s007.docx]

| **Risk factor** | **Outcome category** | **Risk factor category** | **Age-adjusted** | | | | **Multivariable** | | | |
| --- | --- | --- | --- | --- | --- | --- | --- | --- | --- | --- |
|  |  |  | **RR** | **P value** | **95% CI-LOW** | **95% CI-HIGH** | **RR** | **P value** | **95% CI-LOW** | **95% CI-HIGH** |
| **Childhood factors** | | | | | | | | | | |
| **Sex** | B vs. A | Female vs. Male | 0.988 | 0.956 | 0.637 | 1.533 |  |  |  |  |
|  | Mixed vs. A |  | 1.170 | 0.705 | 0.518 | 2.642 |  |  |  |  |
|  | Mixed vs. B |  | 1.185 | 0.684 | 0.524 | 2.680 |  |  |  |  |
| **Breastfeeding** | B vs. A | 7-12 vs. 0-6 m | 1.350 | 0.420 | 0.651 | 2.801 |  |  |  |  |
|  | Mixed vs. A |  | 1.793 | 0.501 | 0.327 | 9.826 |  |  |  |  |
|  | Mixed vs. B |  | 1.329 | 0.745 | 0.240 | 7.367 |  |  |  |  |
|  | B vs. A | >12 vs. 0-6 m | 1.328 | 0.437 | 0.649 | 2.717 |  |  |  |  |
|  | Mixed vs. A |  | 2.995 | 0.190 | 0.580 | 15.466 |  |  |  |  |
|  | Mixed vs. B |  | 2.255 | 0.335 | 0.432 | 11.772 |  |  |  |  |
| **Birth order** | B vs. A | 3^rd^-4^th^ vs. 1^st^-2^nd^ | 1.350 | 0.420 | 0.651 | 2.801 |  |  |  |  |
|  | Mixed vs. A |  | 1.793 | 0.501 | 0.327 | 9.826 |  |  |  |  |
|  | Mixed vs. B |  | 1.329 | 0.745 | 0.240 | 7.367 |  |  |  |  |
|  | B vs. A | >=5^th^ vs. 1^st^-2^nd^ | 1.328 | 0.437 | 0.649 | 2.717 |  |  |  |  |
|  | Mixed vs. A |  | 2.995 | 0.190 | 0.580 | 15.466 |  |  |  |  |
|  | Mixed vs. B |  | 2.255 | 0.335 | 0.432 | 11.772 |  |  |  |  |
| **Any daycare to 3 years** | B vs. A | Yes vs. No | 1.241 | 0.426 | 0.729 | 2.114 |  |  |  |  |
|  | Mixed vs. A |  | 1.406 | 0.474 | 0.553 | 3.574 |  |  |  |  |
|  | Mixed vs. B |  | 1.133 | 0.795 | 0.442 | 2.900 |  |  |  |  |
| ***A. lumbricoides* (tv)** | B vs. A | Yes vs. No | **2.853** | **0.001** | **1.538** | **5.295** |  |  |  |  |
|  | Mixed vs. A |  | 1.077 | 0.916 | 0.272 | 4.266 |  |  |  |  |
|  | Mixed vs. B |  | 0.377 | 0.146 | 0.101 | 1.404 |  |  |  |  |
| ***T. trichiura* (tv)** | B vs. A | Yes vs. No | **2.570** | **0.012** | **1.227** | **5.383** |  |  |  |  |
|  | Mixed vs. A |  | 2.432 | 0.190 | 0.644 | 9.185 |  |  |  |  |
|  | Mixed vs. B |  | 0.947 | 0.931 | 0.274 | 3.265 |  |  |  |  |
| **Any STH (tv)** | B vs. A | Yes vs. No | **3.072** | **<0.001** | **1.798** | **5.251** | **2.815** | **<0.001** | **1.620** | **4.889** |
|  | Mixed vs. A |  | 0.975 | 0.967 | 0.289 | 3.284 | 0.923 | 0.899 | 0.266 | 3.198 |
|  | Mixed vs. B |  | 0.317 | 0.054 | 0.099 | 1.018 | 0.328 | 0.066 | 0.100 | 1.077 |
| **Maternal factors** | | | | | | | | | | |
| **Age (yrs)** | B vs. A | 21-29 vs. <=20 | 1.020 | 0.942 | 0.602 | 1.728 |  |  |  |  |
|  | Mixed vs. A |  | 0.656 | 0.390 | 0.251 | 1.715 |  |  |  |  |
|  | Mixed vs. B |  | 0.644 | 0.369 | 0.247 | 1.681 |  |  |  |  |
|  | B vs. A | >=30 vs. <=20 | 0.803 | 0.478 | 0.438 | 1.473 |  |  |  |  |
|  | Mixed vs. A |  | 0.721 | 0.555 | 0.244 | 2.133 |  |  |  |  |
|  | Mixed vs. B |  | 0.898 | 0.847 | 0.302 | 2.670 |  |  |  |  |
| **Ethnicity** | B vs. A | Non-Afro. vs. Afro. | **0.511** | **0.006** | **0.317** | **0.824** | 0.684 | 0.205 | 0.380 | 1.230 |
|  | Mixed vs. A |  | 0.534 | 0.155 | 0.225 | 1.268 | 1.479 | 0.497 | 0.478 | 4.575 |
|  | Mixed vs. B |  | 1.045 | 0.920 | 0.442 | 2.467 | 2.161 | 0.175 | 0.710 | 6.583 |
| **Education** | B vs. A | Primary vs. Illit | 0.581 | 0.137 | 0.284 | 1.188 |  |  |  |  |
|  | Mixed vs. A |  | 0.396 | 0.131 | 0.119 | 1.316 |  |  |  |  |
|  | Mixed vs. B |  | 0.682 | 0.520 | 0.213 | 2.186 |  |  |  |  |
|  | B vs. A | Second vs. Illit | 0.451 | 0.052 | 0.202 | 1.007 |  |  |  |  |
|  | Mixed vs. A |  | 0.716 | 0.615 | 0.194 | 2.636 |  |  |  |  |
|  | Mixed vs. B |  | 1.586 | 0.479 | 0.443 | 5.679 |  |  |  |  |
| **Maternal STH** | B vs. A | Yes vs. No | **1.888** | **0.005** | **1.216** | **2.930** | **1.603** | **0.042** | **1.017** | **2.529** |
|  | Mixed vs. A |  | 1.194 | 0.678 | 0.518 | 2.751 | 1.228 | 0.645 | 0.513 | 2.940 |
|  | Mixed vs. B |  | 0.632 | 0.281 | 0.275 | 1.455 | 0.766 | 0.546 | 0.322 | 1.823 |
| **Household factors** | | | | | | | | | | |
| **Area of residence** | B vs. A | Rural vs. Urban | 0.772 | 0.308 | 0.469 | 1.269 |  |  |  |  |
|  | Mixed vs. A |  | 0.757 | 0.570 | 0.290 | 1.978 |  |  |  |  |
|  | Mixed vs. B |  | 0.981 | 0.969 | 0.374 | 2.574 |  |  |  |  |
| **Socio-economic status** | B vs. A | Medium vs. Low | 0.809 | 0.458 | 0.461 | 1.418 |  |  |  |  |
|  | Mixed vs. A |  | 0.671 | 0.467 | 0.229 | 1.964 |  |  |  |  |
|  | Mixed vs. B |  | 0.830 | 0.733 | 0.284 | 2.427 |  |  |  |  |
|  | B vs. A | High vs. Low | 0.823 | 0.504 | 0.464 | 1.458 |  |  |  |  |
|  | Mixed vs. A |  | 1.138 | 0.806 | 0.405 | 3.192 |  |  |  |  |
|  | Mixed vs. B |  | 1.383 | 0.536 | 0.495 | 3.863 |  |  |  |  |
| **Monthly income (US$)** | B vs. A | >1 vs. <1 salary | 0.865 | 0.152 | 0.710 | 1.055 |  |  |  |  |
|  | Mixed vs. A |  | 0.933 | 0.696 | 0.657 | 1.323 |  |  |  |  |
|  | Mixed vs. B |  | 1.078 | 0.679 | 0.755 | 1.538 |  |  |  |  |
| **House construction** | B vs. A | Cement/brick vs | 0.785 | 0.386 | 0.454 | 1.357 |  |  |  |  |
|  | Mixed vs. A | Wood/bamboo | 0.528 | 0.183 | 0.207 | 1.351 |  |  |  |  |
|  | Mixed vs. B |  | 0.673 | 0.404 | 0.266 | 1.705 |  |  |  |  |
| **Material goods** | B vs. A | 3-4 vs. 1-2 | 0.985 | 0.946 | 0.632 | 1.535 |  |  |  |  |
|  | Mixed vs. A |  | 0.780 | 0.553 | 0.344 | 1.769 |  |  |  |  |
|  | Mixed vs. B |  | 0.792 | 0.578 | 0.349 | 1.800 |  |  |  |  |
| **Overcrowding** | B vs. A | >=3 vs. <3 pers | 1.236 | 0.353 | 0.791 | 1.931 |  |  |  |  |
|  | Mixed vs. A |  | 1.140 | 0.756 | 0.498 | 2.610 |  |  |  |  |
|  | Mixed vs. B |  | 0.923 | 0.849 | 0.403 | 2.113 |  |  |  |  |
| **Agriculture (tv)** | B vs. A | Yes vs. No | 0.876 | 0.554 | 0.565 | 1.359 |  |  |  |  |
|  | Mixed vs. A |  | 0.856 | 0.712 | 0.375 | 1.953 |  |  |  |  |
|  | Mixed vs. B |  | 0.977 | 0.956 | 0.428 | 2.234 |  |  |  |  |
| **Bathroom (tv)** | B vs. A | Yes vs. No | 0.888 | 0.632 | 0.545 | 1.445 |  |  |  |  |
|  | Mixed vs. A |  | 0.512 | 0.150 | 0.206 | 1.274 |  |  |  |  |
|  | Mixed vs. B |  | 0.577 | 0.222 | 0.239 | 1.395 |  |  |  |  |
| **Peri-domiciliary animals** | | | | | | | | | | |
| **Cats (tv)** | B vs. A | Yes vs. No | 0.798 | 0.543 | 0.386 | 1.651 |  |  |  |  |
|  | Mixed vs. A |  | 1.022 | 0.971 | 0.322 | 3.237 |  |  |  |  |
|  | Mixed vs. B |  | 1.280 | 0.673 | 0.407 | 4.032 |  |  |  |  |
| **Dogs (tv)** | B vs. A | Yes vs. No | 1.097 | 0.821 | 0.492 | 2.447 |  |  |  |  |
|  | Mixed vs. A |  | 0.588 | 0.491 | 0.130 | 2.665 |  |  |  |  |
|  | Mixed vs. B |  | 0.536 | 0.413 | 0.120 | 2.391 |  |  |  |  |
| **Pigs (tv)** | B vs. A | Yes vs. No | 0.902 | 0.695 | 0.539 | 1.510 |  |  |  |  |
|  | Mixed vs. A |  | 1.397 | 0.488 | 0.543 | 3.594 |  |  |  |  |
|  | Mixed vs. B |  | 1.549 | 0.350 | 0.618 | 3.878 |  |  |  |  |
| **Chickens (tv)** | B vs. A | Yes vs. No | 1.014 | 0.947 | 0.675 | 1.523 |  |  |  |  |
|  | Mixed vs. A |  | 0.739 | 0.458 | 0.333 | 1.641 |  |  |  |  |
|  | Mixed vs. B |  | 0.729 | 0.427 | 0.335 | 1.590 |  |  |  |  |
| **Cows(tv)** | B vs. A | Yes vs. No | 0.990 | 0.984 | 0.381 | 2.575 |  |  |  |  |
|  | Mixed vs. A |  | 3.516 | 0.089 | 0.825 | 14.976 |  |  |  |  |
|  | Mixed vs. B |  | 3.550 | 0.073 | 0.890 | 14.159 |  |  |  |  |
| **Horses (tv)** | B vs. A | Yes vs. No | 0.698 | 0.343 | 0.331 | 1.469 |  |  |  |  |
|  | Mixed vs. A |  | 1.100 | 0.897 | 0.261 | 4.626 |  |  |  |  |
|  | Mixed vs. B |  | 1.576 | 0.531 | 0.380 | 6.540 |  |  |  |  |
| **Donkeys (tv)** | B vs. A | Yes vs. No | 0.497 | 0.226 | 0.160 | 1.541 |  |  |  |  |
|  | Mixed vs. A |  | 0.727 | 0.786 | 0.073 | 7.246 |  |  |  |  |
|  | Mixed vs. B |  | 1.461 | 0.747 | 0.146 | 14.639 |  |  |  |  |
| **Mules (tv)** | B vs. A | Yes vs. No | 1.044 | 0.925 | 0.424 | 2.570 |  |  |  |  |
|  | Mixed vs. A |  | 0.605 | 0.656 | 0.066 | 5.515 |  |  |  |  |
|  | Mixed vs. B |  | 0.579 | 0.623 | 0.066 | 5.098 |  |  |  |  |
| **Any equine (tv)** | B vs. A | Yes vs. No | 0.754 | 0.421 | 0.379 | 1.500 |  |  |  |  |
|  | Mixed vs. A |  | 0.954 | 0.947 | 0.237 | 3.843 |  |  |  |  |
|  | Mixed vs. B |  | 1.265 | 0.737 | 0.320 | 5.004 |  |  |  |  |

**S3 Table: Age-adjusted and multivariable effects of childhood, parental, and household factors on the relative risk of infections with *G. lamblia* assemblages A vs. B and mixed (A and B) vs. A or B.**

Age-adjusted analyses include polynomial terms for age up to power of 5 (all P<0.001). RR – relative risk. CI – confidence interval. Time varying (tv) variables. Data on household factors were collected around the time of birth of the child unless specified as tv. Material goods – number of household electrical goods. Pigs, chickens, cows, and equines – keeping these animals around house. Agriculture – child lives on a farm or visits a farm at least once a week. STH – soil-transmitted helminth infection. Non-Afro – non-Afro-Ecuadorian. yrs – years. m – months. SES -socioeconomic status. Overcrowding – persons/sleeping room. Statistically significant findings (P<0.05) are shown in bold.
