# Supplementary material for: Epidemiology of giardiasis and assemblages A and B and effects on diarrhea and growth trajectories during the first 8 years of life: Analysis of a birth cohort in a rural district in tropical Ecuador
Source: PLoS Negl Trop Dis. 2023 Nov 20;17(11):e0011777. doi: 10.1371/journal.pntd.0011777 (PMC10695370; doi:10.1371/journal.pntd.0011777)
Supplement: S4 Table — RR–relative risk; OR–Odds ratio; CI–confidence interval; Y–yes; N–no; X–interactions. Statistically significant findings (P<0.05) are shown in bold. (DOCX) [file pntd.0011777.s008.docx]

| Giardia assemblage | **Height-for-age z-scores** | | | | **Weight-for-age z-scores** | | | | **BMI-for-age z-scores** | | | | **Diarrhea** | | | |
| --- | --- | --- | --- | --- | --- | --- | --- | --- | --- | --- | --- | --- | --- | --- | --- | --- |
|  | Estimate | P value | 95% CI | | Estimate | P value | 95% CI | | Estimate | P value | 95% CI | | RR/OR | P value | 95% CI | |
| **B vs. A** | 0.028 | 0.676 | -0.103 | 0.160 | 0.059 | 0.318 | -0.057 | 0.176 | 0.061 | 0.430 | -0.091 | 0.213 | 0.896 | 0.748 | 0.457 | 1.757 |
| **Mixed vs. A** | -0.066 | 0.625 | -0.332 | 0.200 | -0.037 | 0.756 | -0.270 | 0.196 | 0.036 | 0.815 | -0.266 | 0.338 | 1.435 | 0.571 | 0.412 | 4.994 |
| **Mixed vs. B** | -0.094 | 0.463 | -0.346 | 0.157 | -0.096 | 0.392 | -0.317 | 0.124 | -0.025 | 0.863 | -0.310 | 0.260 | 1.602 | 0.432 | 0.494 | 5.193 |

**S4 Table: Effects of *G. lamblia* assemblage comparisons (among those with *G. lamblia* infection) on probability of diarrhea and on trajectories for height-for-age, weight-for-age, and body mass index (BMI)-for-age z scores.**

RR – relative risk; OR – Odds ratio; CI – confidence interval; Y – yes; N – no; X – interactions. Statistically significant findings (P<0.05) are shown in bold.
