# Supplementary material for: Epidemiology of giardiasis and assemblages A and B and effects on diarrhea and growth trajectories during the first 8 years of life: Analysis of a birth cohort in a rural district in tropical Ecuador
Source: PLoS Negl Trop Dis. 2023 Nov 20;17(11):e0011777. doi: 10.1371/journal.pntd.0011777 (PMC10695370; doi:10.1371/journal.pntd.0011777)
Supplement: Attachment — Submitted filename: Point-by-pont response to reviewers_Giardia_FINAL.docx [file pntd.0011777.s010.docx]

**Point-by-point response to Reviewers’ comments**

Comment 1:……Giardiasis, caused by the enteric protozoal parasite Giardia lamblia (also known as Giardia
86 duodenalis and Giardia intestinalis), is a neglected tropical disease with a worldwide
87 distribution [1] .
This comment is not valid any more, pl rephrase and update. Giardia is everywhere, it is not tropical, it is partially neglected, all of these neglected diseases are now top priority.
Also, you need to emphasize the role of drinking water and outbreaks transmission of G., update and take lit reviews in consideration. Same for vegetables contamination and transmission. (Introduction and discussion). 

Response 1: We have corrected the text to state, “Giardiasis, caused by the enteric protozoal parasite Giardia lamblia (also known as Giardia duodenalis and Giardia intestinalis), is a neglected infectious disease with a worldwide distribution”. We have included an additional reference for a recent review of transmission of G. lamblia (reference number 8). Outbreaks transmission in non-endemic settings is mentioned in the Discussion (lines 634-636).

Comment 2: 101 endemic settings is less clear [17].Giardiasis is considered to cause stunting and/or failure-to-..
Pl keep space between after the dot.

Response 2: Corrected.

Comment 4: 142 temperatures generally ranging 23-32oC with yearly rainfall of around 2000-3000mm.
Keep space between units and words.

Response 4: Corrected

Comment 5: L 177-184, keep space between units and words.
Same in the following chapters

Response 5: Corrected.

Comment 6: Question: what about microscopical analysis results? please, provide explanation for this

Response 6: We used qPCR to detect G. lamblia infections because microscopy is poorly sensitive in comparison (for example, reference 29). Microscopy was only used for the systematic detection of STH ova and larvae.

Comment 7: Question How about other assemblages. Please, provide explanation for this.

Response 7: Assemblages A and B are the dominant assemblages observed in Latin American with other assemblages being observed infrequently in human fecal samples (references 15 & 16). Sanger sequencing of G. lamblia assemblages in the present study only detected assemblages A and B (lines 369-373).

Comment 8: Usually, three loci are using to detect and characterize Giardia. This is a common agreement in the science of Giardia

Please, provide explanation for this

Response 8: We used molecular methods (qPCR) targeting a single locus to detect *G. lamblia* infections and this approach is widely used in the literature and considered to provide valid findings (reference 29). Positive samples by qPCR were characterized at the assemblage level: 18S rRNA gene has a multicopy nature, which gives it greater success in PCR amplification when using samples with a small amount of DNA and a greater amount of PCR inhibitors such as fecal or environmental samples (Minetti et al., 2015) and its efficient use to determine Giardia assemblages has been previously reported (reference 33; Asher et al., 2014; Read et al., 2002) We agree with the reviewer that three loci are generally used to characterize *G. lamblia* to subassemblage level. However, in the present study we wished to characterize positive samples just at the assemblage level for which we targeted single loci using two methods as reported by others (reference 46; Kohli et al., 2008; Mohammed Mahdy et al., 2009). Full reference details are provided below.


Comment 9: 300 dependent risk of giardia infections predicted by the longitudinal models against observed
Pl correct species name
Linguistic issues and grammar corrections are needed

Response 9: giardia has been substituted by *G. lamblia* in the text. The manuscript text has been reviewed and edited by a native English speaker.

Comment 10: References
Pl revise, correct and unify the style of cited references. There are irregularities.

Response 10: Style of cited references has been corrected.

Comment 11: Page 10, line 169ff: I am not sure how this relates to Giardia or other protozoal diagnostics? Were there only STH detected or “all” parasites. Please include a statement for clarification.

Response 11: Only STH were detected by microscopy. We have added the following to the text: “A positive sample for STH was defined by the presence of..”

Comment 12: Page 10, line 178: How much stool sample was processed? Was fresh stool or preserved stool or both processed?

Response 12: The following has been added to the text: “50 mg of stool stored in 90% ethanol at -30°C” (line 200).

Comment 13: Page 10, line 181/182: Please provide more information about the qPCR assay: Provide primer/probe sequences, please include statement why the assay was only using 7µl total volume (usually 25µl are used), was there a inhibition control PCR included (if not, why not?), check primer concentration (also in the other PCR assays)

Response 13: Primer/probe sequences for the molecular assays are provided now in the supplement (S1 table). In our first Ecuador qPCR publication (reference 29), we showed the use of 7 µl total volume worked equally well as the standard volume of 25 µl, likely due to running each test as a single-plex assay thus allowing sufficient DNA polymerase to amplify the single target. A TaqMan-based internal amplification control was included during the DNA extraction stage and then amplified by qPCR. We have added the following sentence to the text (lines 208-217): “All extractions included 1 ul of internal amplification control (IAC) plasmid at a concentration of 100 pg/ul, containing a unique 198-bp sequence and detected by qPCR using PCR primers and probe sequences as described [30]”. Primer concentrations have been reviewed for all PCR assays. Our master mix were prepared to work with 100 reactions to use with 96 well plates.

Comment 14: Page 11, line 188ff: Please include primer and probe sequences of the newly developed assay. What are the parameters of the new assay (analytical sensitivity and specificity)? What was the positive/negative control DNA for the assay?

Response 14: Primer and probe sequences for all 3 PCRs are now provided in a new supplementary table (S1 Table). The positive control was derived from samples found to be positive by qPCR and then sequenced as assemblage A or B. Positive controls for A and B and negative controls (without template) were included in all reactions. At >28.02 fg/ul (equivalent to a Ct value of 33 in this assay) or higher parasitic loads in the samples, we estimate 100% sensitivity and specificity for the 18S rRNA SNP Genotyping Assay for assemblages A and B (i.e none of the qPCR-negative samples amplified generated a positive result and allelic discrimination plots were indeterminate; all samples with DNA >28.02 fg/ul gave a positive result for assemblages A and/or B).

Comment 15: Page 11, line 197: what is “assay working stock”, please specify.

Response 15: The term “assay working stock”, has been substituted by “custom TaqMan® SNP Genotyping Assay solution (Applied Biosystems) for the SNP TaqMan assay”. This solution contained the primers and probes for the assay.

Comment 16: Page 12, Line 219-221: Well, that is not as simple as Giardia represents a tetraploid parasite with assemblage B showing a high proportion of allelic sequence heterogeneity (also shows double peaks in chromograms). Please clarify.

Response 16: We agree with the reviewer that assemblage B has significant allelic sequence heterogeneity. However, Sanger sequencing was done using a highly conserved locus that only showed single peaks in the regions of sequence heterogeneity used to identify assemblages. These mixed sequencing profiles were characterized by a clean sequence up to a point where a nucleotide difference occurred between the 2 templates. In the case of a substitution, 2 bases were present in the same position (these double peaks are present at the position characterizing each genotype A and B) and where there was an insertion or deletion, it generates 2 overlapping sequencing profiles (reference 33). In this sense, the profile found in the electropherograms of the samples identified as mixed infected was conserved among all these samples. Further, all samples showing mixed infections using Sanger sequencing were confirmed using the SNP Genotyping assay (a statement to this effect has been added to Methods [lines 260-262]).

Comments 17: Reviewer #1: The results are overall well presented. However Table 3 lacks information in order to be readable on its own. For instance, age2 and age3 are not explained in the footnotes.
See also below.

Response 17: We have clarified the legend for Table 3 as requested.

Comment 18: Page 15, line 297-301: I found it difficult to follow the numbers. Please also include a supplementary table with all PCR data for each child, so that interested readers maybe able to use the data afterwards. Also make statement whether children stay Assemblage A or B positive or how many children show different assemblages in different longitudinal samples, etc. There is much more information in the dataset as now shown. Also interesting: Had the albendazole treatment some influence on the Giardia-positivity rates (Albendazole is an alternative Giardia treatment) .

Response 18: We have provided the data file as a supplementary file for interested readers to review and/or analyse. We have added a new Figure showing the proportion of children with assemblage data who maintain the same assemblage (A or B) detected during follow-up and those who switch assemblages (A to B or B to A) in S2 Fig. Anthelmintic treatment was excluded from Table 1 as this variable had no effect on *G. lamblia* infection status during follow-up. We have now included this variable in Table 1.

Comment 19: Page 20, line 401-406: is this maybe an indication for zoonotic transmission of Ass B? Did the authors investigate the subtypes of assemblage AI and AII (AII being only found in humans, whereas AI might also be zoonotic)

Response 19: Possibly at subassemblage level - we did not get a signal at assemblage level with any of the peri-domiciliary animals included in the analysis in Table 3. Our analytic approach only allowed determination at assemblage level.

Comment 20: Fig. 4: The Y axes should be risk of developing diarrhea, right? Please check.

Response 20: Correct. This has now been changed in the revised Fig 4.

Comment 21: Reviewer #1: The discussion and conclusion is mostly well balanced. There could be more caution on small sub-groups and long time distance between sampling for some interpretations.

Response 21: We have added statements to study limitations for sub-groups with small samples (lines 722-723) and have evaluated potential effect of extended time between routine sampling times through the sensitivity analysis described in lines 537-546 (and illustrated in S4 Fig).

Comment 22: Reviewer #2: The complex epidemiological situation with Giardia offer space for myn discussions. However, the authors commented and discussed their results in accurate manner

Response 22: We thank the reviewer for his/her positive comments

Comment 23: Reviewer #3: PH is adressed and conclusions are not over-interpreted.
Discussion is sufficient and limitations are described.

Response 23: We thank the reviewer for his/her positive comments

Comment 24: Page 24, line 485: “B” should probably mean “A”?

Response 24: Corrected.

Comment 25: Maybe add a paragraph for assemblage B being the dominating assemblage type. What is the reason? 

Response 25: We have added additional text to the discussion discussing why assemblage B may be dominant in this setting based on the analysis of factors associated with relative risk of assemblage B vs. A (lines 606-610).

Comment 26: How do the results relate to the Giardia data of the GEMS study (Kotloff, K.L., et al., The Lancet, 2013. 382(9888): p. 209-222.)?

Response 26: The GEMS, a landmark case-control study of children aged up to 5 years presenting with moderate-to-severe diarrhoea in several settings in Africa and Asia, showed no significant associations between *G. lamblia* infection (detected by immunoassay) with this outcome. There was some evidence of an inverse association in the 2^nd^ year of life (OR 0.61, 95% CI 0.22-1.2) but not in other age groups. The findings of the GEMS study are therefore consistent with the findings of a meta-analysis of observational studies as detailed in the Discussion (lines 634-640).

Comment 27: Reviewer #3: Minor points - There are some spelling errors that should be checked, eg.:
Page 4 line 56, there is an additional “in” before “coastal”; 
page 5, line 63, “which” should be probably removed; 
page 6, line 89 assemblies” should be “assemblages”;

Response 27: Corrected except for “which” on page 5, line 63, that we prefer to keep unchanged for the sense of the sentence. 

Response 28: While the first sentence in Results in the abstract focuses on total number of stool samples, 2812, this obscures the fact that no time points had more than 376 delivered stool samples, and at 1 month only 102. This has implications for the interpretation of results. Comments on this is needed.

Response 28: We agree that relatively few samples were collected at 1 month although infection rates were negligible at this age (1 infection detected in 102 samples collected [Table S1]). Detailed samples numbers are provided in the Results (lines 367-369) including median (Q1-Q3) [6 (5-8) samples] for valid observations/samples collected from the children, as well as observed and predicted infection rates (Table S1). We appreciate the difficulty of presenting longitudinal data in the presence of missing observations. These are complex data, and our analyses properly addressed the challenges of missing observations by employing mixed models methodology for data analyses which correctly account for all complete observations regardless of imbalances in counts at each time point. The analytic strategy used considered the hierarchical longitudinal structure of the observations – with ‘between and within’ children’s sources of variability and which operated under specified assumptions on missing data, depending on the GEE or random effects approach. This is explained in the section on statistical analytic strategy. The are several reasons why we opted for this approach rather than using simple comparative tests. One reason was the observational nature of the study - this was not a simple randomized study and the differences between samples at different time points could be potentially explained by multiple explanatory variables as derived in multivariable models (Table 2). Most importantly, any within-group tests would have only considered children with complete observations at all time points, during the whole of follow-up, resulting in loss of information. The assumptions on the missing data cannot be tested from these data (details can be found in for example in: Ibrahim and Molenberghs, 2011) but both missing at random and missing completely at random assumptions assume that the missing data are ignorable in a statistical sense. However, the main advantage of using our analytic approach was to make full use of all observations. We did sensitivity analyses for the risk factors analysis using extreme case scenarios such as considering all missing outcomes indicating infection or otherwise, and these showed similar qualitative findings as to those obtained using all observations.

# Comment 29: From the publication which the authors say describe the overall design and details of the study, altogether 2244 children had been followed up to 3 years. Still, the authors only included 504 children recruited in 2008 and 2009. I don’t see the rationale for this, since more participants would have strengthened the conclusions. Please explain.

Response 29: The children included were those in a sub-sample of 504 cohort children included in a surveillance cohort for diarrhea.

Comment 30: The authors include the presence of STH in their analysis, while other pathogens are not considered. This is particularly important when analysing the relationship between Giardia and diarrhea episodes. One further clarification is needed in this context; is the question of diarrhea only a question of present diarrhea at the time of sampling, or is it a question of diarrhea during a period of time. Since the authors have extracted DNA/RNA, have other pathogens been analysed? The presence of other common pathogens could potentially confound the role of Giardia.

Response 30: Stool samples for diarrhoea represent samples taken during a diarrheal episode as defined in Methods (lines 171-172). We analysed the effects childhood, maternal and household STH infections but not those of other pathogens. We have included the lack of data for other diarrheal pathogens as potential uncontrolled confounders in study limitations (lines 713-715).

Comment 31: Assemblage comparisons: As far as I can understand, approximately 600 samples could be genotyped. Could seemingly lack of importance of either genotype be explained by the paucity of available genotyping at each time point?

Response 31: The reviewer is correct that a lack of observed associations could be explained by lack of power as discussed in study limitations (lines 721-724).

Comment 32: Follow-up time points: Up to the age of 3 years the children are seen/give stool samples every 6 months. Even six months intervals are long intervals when investigating the role of Giardia in stunting, and interpretations should be cautious. But after the age of 3, the next sample comes two years later, and then 3 years after that again, at 8 years. While up to 3 years this study confirm previous trials, I think interpretations on the role of Giardia after this time is weak and could be omitted.

Response 32: Previous studies have used more frequent stool sampling to study the effects of *G. lamblia* infection on diarrhea and growth to 3 years of age (references 21-23). Despite less frequent sampling in the present study, we were able to observe effects of *G. lamblia* infection on both outcomes in this study setting. The lack of effect, for example, on growth after 4 years of age might be explained by infrequent sampling (i.e. leading to a null effect) but because we believe this observation to be valid for several reasons: i) it is consistent with the findings of a previous longitudinal study (reference 23); ii) it is biologically plausible as effects on growth are likely to be most marked in earlier than later childhood because of the greater growth velocity in early childhood that is followed by a stabilization of growth rates; and iii) the risk of infection at 5 and 8 years is likely to be strongly associated with risk of infection at any time between during the periods 3-5 years and 5-8 years, respectively, because of continuing exposures to environmental risk factors that largely determine infection risk independent of any individual susceptibility. We agree with the reviewer that more frequent stool sampling times would likely give us more accurate results but that does not necessarily make the findings beyond 3 years ‘weak’. In fact, in this analysis, the data up to 3 years ‘feeds’ into later observation times and shapes the age-dependent trends in infection risk and their effects on health outcomes. The statistical analytical methodology applied here (non-linear mixed modelling) makes full use of all complete growth observations as well as their implicit within-subject correlations through time, regardless of the evenness of time intervals at which they were taken. The analyses account for these uneven intervals: the further apart the measurements, the smaller the correlations. The sensitivity analyses, with similar models applied to data restricted to measurements up to 3 and 5 years, respectively, match the subsequent trends which result using data up to 8 years – with some gain in efficiency, namely smaller standard errors (S4 Fig). We believe it is a valid approach to use all the relevant epidemiological and laboratory data collected that provide novel insights into the changes over time in *G. lamblia* infection and effects of *G. lamblia* on the health of children in a relatively neglected population. We now include text for these sensitivity analyses in the Results (lines 537-546) with the new supplementary Figure (S4 Fig).

Comment 33: I provide my comments above
i would like to suggest the publication of the paper based on useful results and data after the major issues are commented

Response 33: We thank the reviewer for his/her positive comments

Additional references

Asher, A. J., Holt, D. C., Andrews, R. M., & Power, M. L. Distribution of Giardia duodenalis Assemblages A and B among Children Living in a Remote Indigenous Community of the Northern Territory, Australia. PLOS ONE 2014:*9*(11), e112058.

Ibrahim JG, Molenberghs G. [Missing data methods in longitudinal studies: a review.](https://pubmed.ncbi.nlm.nih.gov/21218187/) Test (Madr). 2009;18(1):1-43.

Kohli, A., Bushen, O. Y., Pinkerton, R. C., Houpt, E., Newman, R. D., Sears, C. L., Lima, A. A. M., & Guerrant, R. L. Giardia duodenalis assemblage, clinical presentation and markers of intestinal inflammation in Brazilian children. Trans R Soc Trop Med Hyg. 2008; *102(7), 7*18–725.

Maloney, J. G., Molokin, A., & Santin, M. Assessment of next generation amplicon sequencing of the beta-giardin gene for the detection of Giardia duodenalis assemblages and mixed infections. Food Waterborne Parasitol. 2020: 21, e00098.

Minetti, C., Lamden, K., Durband, C., Cheesbrough, J., Fox, A., & Wastling, J. M. Determination of Giardia duodenalis assemblages and multi-locus genotypes in patients with sporadic giardiasis from England. Parasit Vectors, 2015: *8*(1), 444.

Mohammed Mahdy, A. K., Surin, J., Wan, K. L., Mohd-Adnan, A., Al-Mekhlafi, M. S. H., & Lim, Y. a. L. Giardia intestinalis genotypes: Risk factors and correlation with clinical symptoms. Acta Tropica 2009:112(1), 67–70.

Read, C., Walters, J., Robertson, I. D., & Thompson, R. C. A. Correlation between genotype of Giardia duodenalis and diarrhoea. Int J Parasitol 2002: 32(2), 229–231.
